# A Rare Case of Multifocal Giant Cell Tumor of the Right Hand Little Finger Flexor Tendon Sheath

**DOI:** 10.7759/cureus.86094

**Published:** 2025-06-15

**Authors:** Jason Yew Hung Ling, Sei Haw Sem

**Affiliations:** 1 Orthopedics and Traumatology, Hospital Universiti Kebangsaan Malaysia, Kuala Lumpur, MYS; 2 Orthopedics, Hospital Kuala Lumpur, Kuala Lumpur, MYS

**Keywords:** flexor tendon, giant cell tumor, hand, surgical excision, tendon sheath

## Abstract

Giant cell tumor of the tendon sheath (GCTTS) or nodular tenosynovitis arises from a discrete solitary nodule in the tendon sheath of finger joints. Multifocal GCTTS is a rare entity, whereby its etiology is not fully understood and is different from the diffuse type of GCTTS. We report a case of multifocal GCTTS along the tendon sheath of the flexor digitorum profundus of the little finger. Surgical excision was done with no signs of recurrence upon one-year follow-up.

## Introduction

Giant cell tumor of the tendon sheath (GCTTS) is the second most common soft tissue tumor in the hand after ganglion cyst [1]. Most of the time, it is solitary, involving the palmar aspect of the radial three digits and near the distal interphalangeal joint area. It is also a slow-growing benign tumor, presenting insidiously as a firm and painless swelling over the years. In contrast to GCTTS, pigmented villonodular synovitis (PVNS) typically presents as a diffuse condition affecting larger joints of the lower limb. The diffuse form is associated with joint pain and effusion. Ganglion cysts, on the other hand, most commonly occur on the dorsal aspect of the wrist, are fluctuant, and demonstrate positive transillumination. GCTTS is primarily managed with surgical excision. In cases of diffuse PVNS, treatment usually involves synovectomy, often combined with radiation therapy [2]. Ganglion cysts are generally treated conservatively, with surgical excision reserved for cases that cause pain or functional impairment. Multifocal GCTTS is rare. We report a case of GCT of flexor tendon sheath, which is unusually large and multifocal, spreading across the wrist joint proximally into the distal aspect of the forearm.

## Case presentation

**Figure 1:**
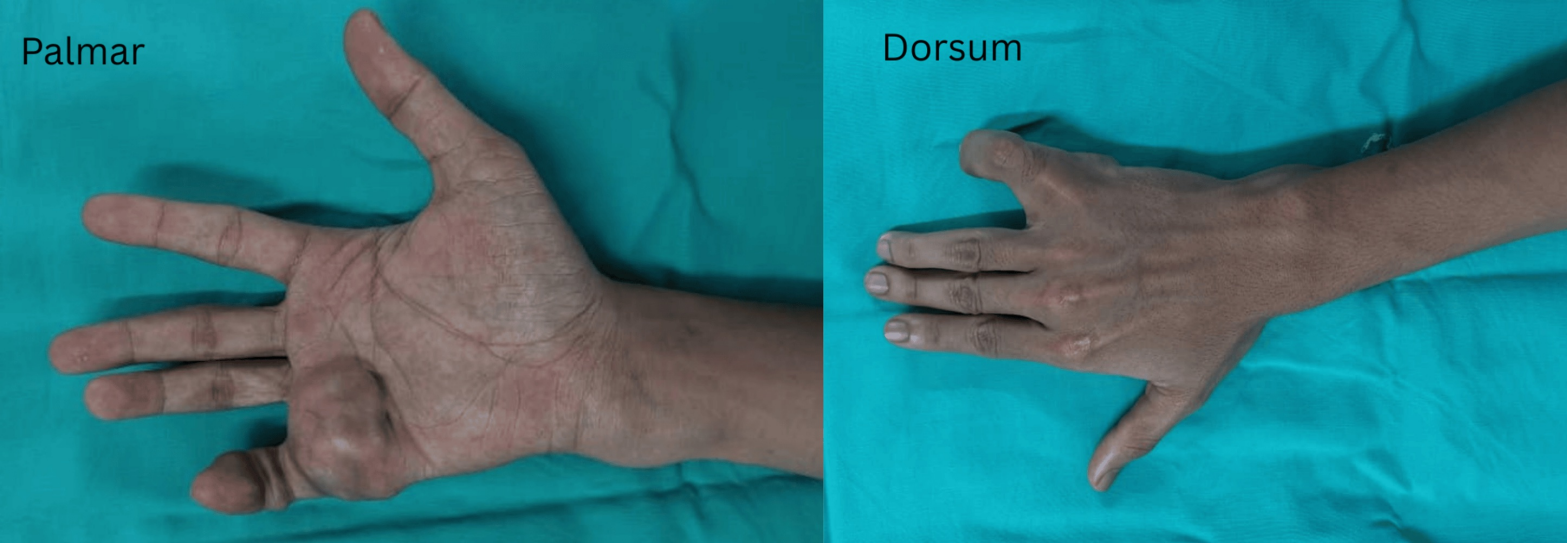
Clinical photos of the right hand Multiple lobulated swellings over right little finger, palmar ulnar border of right hand and distal aspect of right forearm region

A 19-year-old gentleman, right-hand dominant, presented to us in August 2022 with multiple painless swellings over the palmar aspect of his right hand, gradually increasing in size over two years, affecting his grip strength. He denied any constitutional symptoms, fever, or history of trauma. Clinical examination revealed multiple, firm, lobulated, and well-circumscribed swellings along the flexor aspect of the right little finger, the palmar region of the hand, and the distal ulna aspect of the right forearm as shown in Figure 1.

**Figure 2:**
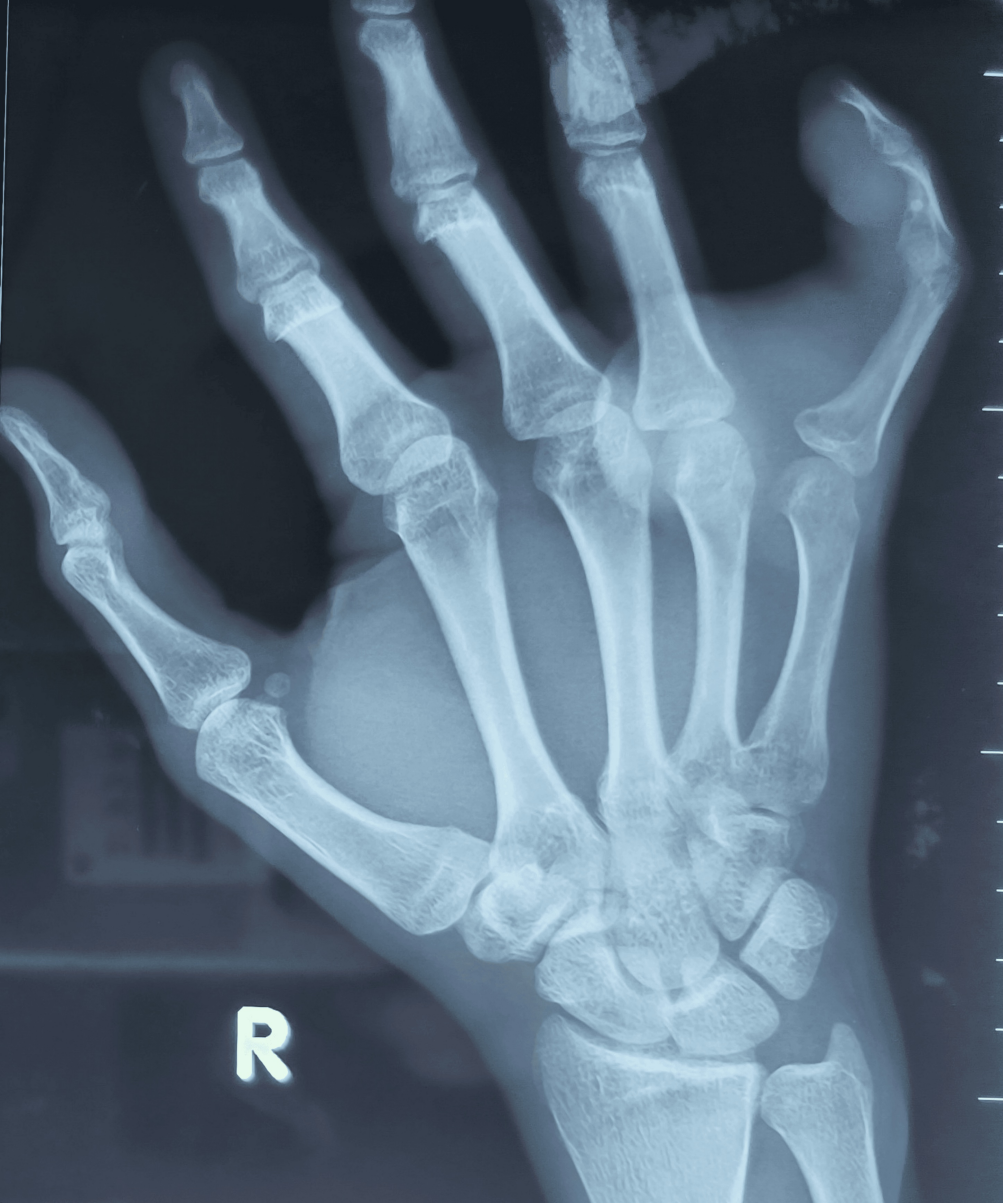
Plain X-ray of the right hand X-ray of the right hand showed significant soft tissue swelling but no sign of bony indentation or periosteal reaction

His little and ring fingers' sensations were intact. All finger circulation was normal. The range of motion of his right hand's little finger distal interphalangeal joint (DIPJ), proximal interphalangeal joint (PIPJ), metacarpophalangeal joint (MCPJ), and the wrist was 20-45°, 20-90°, 0-10°, and 0-30°, respectively. As for the contralateral side, his DIPJ, PIPJ, and MCPJ ranges of motion were 0-90°, and his wrist range was 0-60°. Grip strength power was Medical Research Council (MRC) grade 4 for his right hand. X-rays radiograph (Figure 2) was done to exclude any bony involvement. 

**Figure 3:**
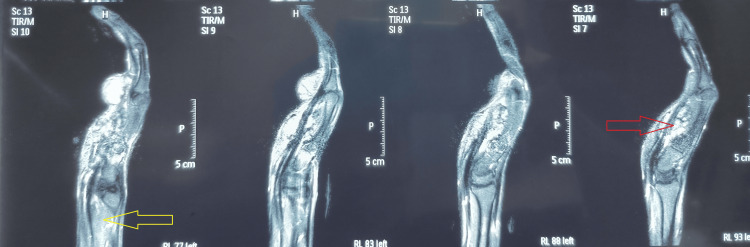
MRI right hand T1-weighted imaging post contrast MRI sagittal view showed the extent of the multilobulated giant cell tumor flexor tendon sheath, invading the mid-carpal space (as shown by the red arrow), crossing the wrist joint into the distal ulnar aspect of the forearm (as indicated by the yellow arrow)

Ultrasound and MRI (Figure 3) scans showed diffuse infiltrative soft tissue masses and synovial thickening over the flexor tendon sheath. 

**Figure 4:**
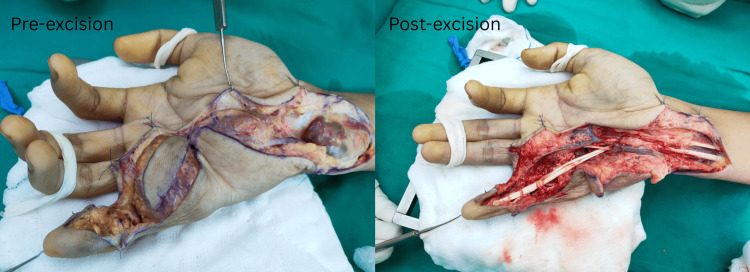
Intraoperative photos The image above shows our approach to the excision of the multifocal giant cell tumor of the tendon sheath via Brunner's incision

**Figure 5:**
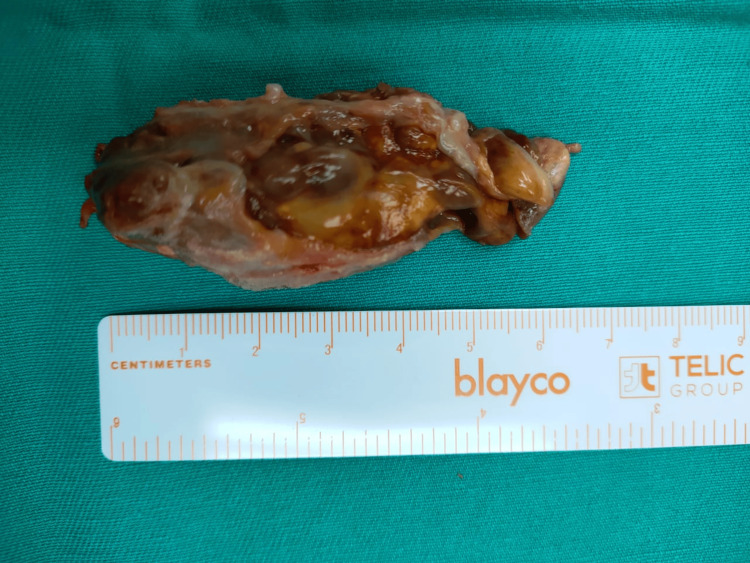
A completely excised tumor The largest tumor measuring 6.5 cm x 2 cm, which is brownish/yellowish in color, consistent with the diagnosis of GCTTS

The complete blood count showed a total white count of 7.7 x 10^9^/L. His C-reactive protein (CRP) and erythrocyte sedimentation rate (ESR) were 1.3 mg/dL and 8 mm/hr, respectively. Autoimmune markers such as the rheumatoid factor, anti-ccp, C3/C4, and dsDNA were negative. Excision biopsy was done via Brunner’s incision under general anesthesia with magnification loupes. Intraoperatively, extensive multiple yellowish-brown tumors were noted arising from the flexor digitorum profundus (FDP) tendon sheath (Figure 4) over the distal ulna aspect of the right forearm, zone 2-3 flexor aspect of the hand, with extension into the mid-palmar space and along the little finger flexor tendon sheath with signs of indentation to the proximal phalanx. The largest tumor measured 6.5 cm x 2 cm over the ulnar palmar aspect of the right hand (Figure 5).

**Figure 6:**
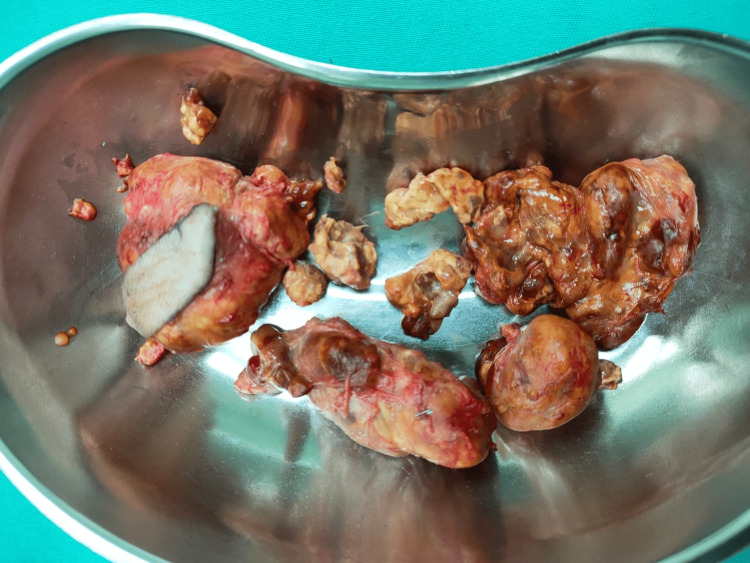
Macroscopic photo of the tumors A total of seven discrete nodules of different sizes were excised as shown in the above figure. Histopathological sampling was sent

The operation was uneventful, and numerous tumors were removed (Figure 6).

**Figure 7:**
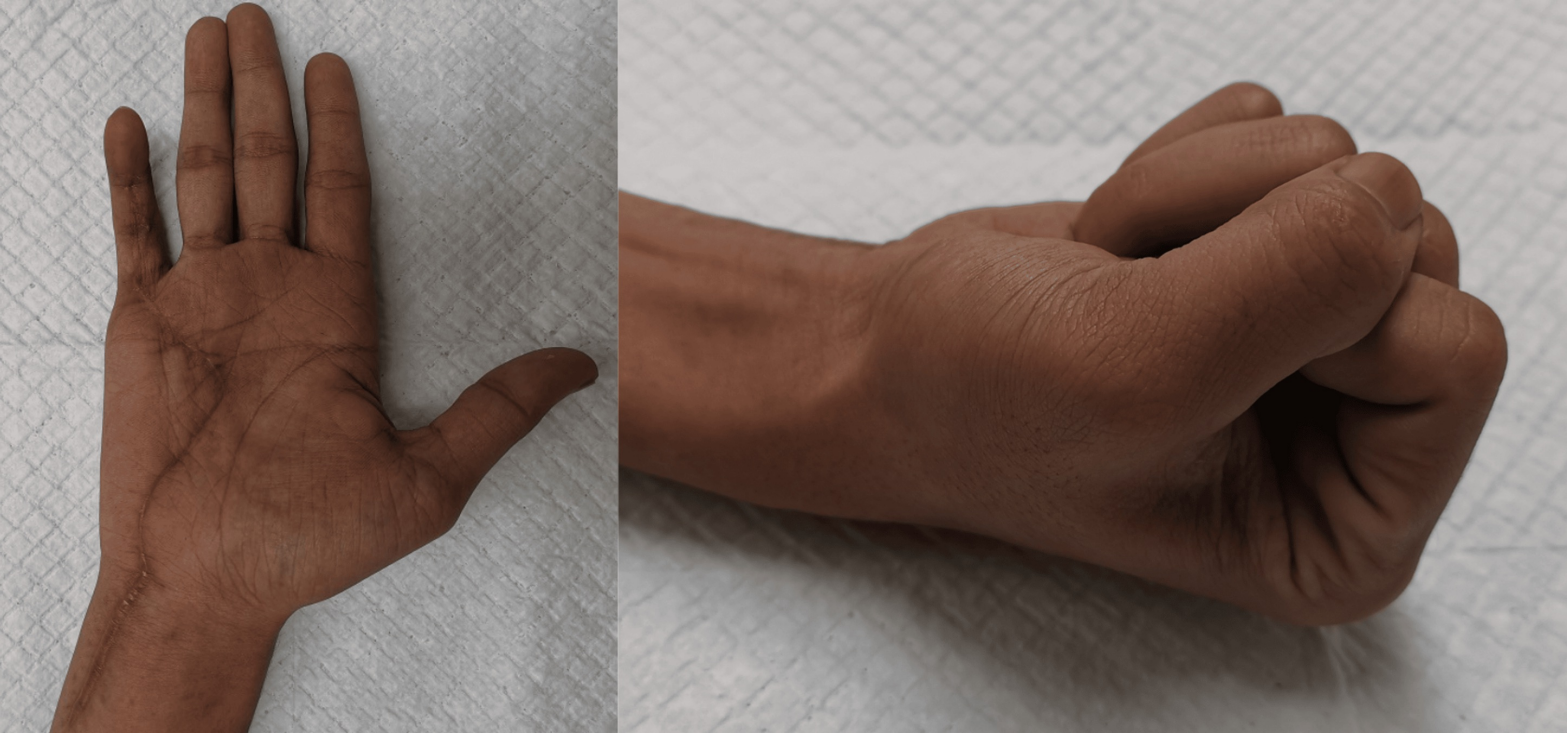
Clinical photo post-excision surgery two months The image shows the patient is able to fully extend and flex his hand at the level of the DIPJ, PIPJ, and MCPJ. Scars are well-healed with no signs of contracture

Histopathological examination revealed multinucleated giant cells scattered along the mononuclear cells containing round to oval vesicular nuclei and proliferating histiocytes. Under microscopic examination, there was the presence of hemosiderin, and the observed mitotic count was 4/10 HPF with no aberrant forms. During follow-up postoperatively, his wound was well-healed, and his right hand regained full power grip two months later (Figure 7) with intact neurovascular status. Range of motion over the right hand little finger DIPJ, PIPJ, MCPJ, and the wrist was 10-70°, 0-90°, 0-90°, and 0-60°, respectively.

The patient was able to gradually return to his work six weeks after surgery. There was no evidence of recurrence up to a year.

## Discussion

GCTTS is a benign soft tissue tumor of the hand and originates from the synovium of the tendon sheath (nodular type) or large joint (diffuse type). It usually presents at the third to fifth decades of life. GCTTS is the second most common benign tumor in the hand, comprising about 15-27.5% of cases [1]. Ganglion cysts are the most common, accounting for approximately 65% of all masses in the hand [3]. Although the etiology of GCTTS is not clearly known, it is thought to be due to a mutation in the stromal cells of the synovial membrane that causes the colony-stimulating factor 1 to be overexpressed. This overexpression attracts cells of the mononuclear phagocyte lineage that express CSF1R into the tumor mass [4].

Some of the differential diagnoses for GCTTS may include ganglion cyst, pigmented villonodular synovitis, fibroma, desmoid tumor, and even benign bone tumor. Therefore, it is crucial to be able to identify the key differences of GCTTS from the rest through clinical judgement and radiological imaging. GCTTS usually presents as a painless mass or swelling near a joint or tendon sheath, most commonly in the hand. As the tumor progresses, it may cause pain, joint stiffness, and even functional impairment. A ganglion cyst appears as a fluid-filled mass that may be associated with joints or tendons. From MRI, ganglion cyst typically demonstrates a well-defined, cystic appearance, whereas GCTTS often exhibits solid components, with areas of low signal intensity representing hemosiderin deposition. It also assesses the extent of tumor involvement, including extra-articular extension, which is important for preoperative planning [5]. Pigmented villonodular tumor of tendon sheath (PVNS), a related condition, can also share radiological features, but typically, PVNS is more diffuse and involves the joint synovium extensively, whereas GCTTS is typically localized to tendon sheaths.

In a paper published by Al-Qattan [6] in 2001, GCTTS was classified into type I (entire tumor surrounded by one pseudocapsule) and type II (entire tumor not surrounded by one pseudocapsule). In our case, it can be classified as type IIC, which is a multicentric type with separated, discrete lesions in the same digit. Type II tumor has a recurrence rate of about 38%. The recurrence rate is influenced by factors such as the presence of satellite lesions, incomplete excision, degenerative joint diseases, involvement of the distal interphalangeal joint, mitotic activity on histology, gene nm 23, and radiographic evidence of pressure erosion [7-8]. Interestingly, a study by Ozben et al. [9] has shown that the only significant risk factor is joint capsule adjacency of the tumor, especially over the interphalangeal joints. Tumors that involve more than one site, such as type IIC, may be more difficult to completely excise, which leads to higher chances of recurrence.

Surgical excision remains the primary treatment for GCTTS. Goals of treatment for GCTTS include complete excision of the lesion, minimizing the chance of recurrence, and maintaining normal hand function. Extra attention has to be paid during surgical dissection to minimize the chances of recurrence. As the recurrence rate is 45% [10], it is crucial to be meticulous during soft tissue dissection. In our case, the tumor has extended into mid mid-palmar space and the distal aspect of the forearm, which was extremely rare. Fortunately, there was no significant neurovascular compromise from the expansile nature of the tumor itself.

Surgical field exposure and dissection can be extremely challenging to preserve the existing function of a patient’s hand. Microsurgical techniques and magnification may improve visualization and aid in achieving total resection, especially when the tumor is adherent to tendons, joints, or neurovascular structures. Tumors that have caused bone erosion or significant changes to local anatomy may require additional procedures such as bone grafting or even joint reconstruction to rectify the damage. Adjuvant therapy, such as radiation therapy, can also be considered in recurrent or infiltrative GCTTS cases to provide local tumor control with preservation of hand function [11].

## Conclusions

This case report describes an exceptionally rare presentation of multifocal GCTTS in the right hand. Uniquely, the tumor involved the flexor digitorum profundus tendon of the little finger and extended proximally, crossing the wrist joint into the distal forearm. Unlike the typical solitary, localized GCTTS, this multifocal and extensively spreading variant has not been previously reported. The case emphasizes the need for meticulous surgical dissection, especially in multifocal and extensive cases, to ensure complete tumor removal and minimize recurrence risk while preserving hand function. However, the limitations in our case include a short follow-up duration of only one year and the absence of histological slide sections, which could have provided stronger academic references. Given the substantial recurrence rate associated with GCTTS, ongoing clinical surveillance is critical in managing such patients.
